# Hepatitis B Virus Infection in Tanzania: Current Status and Challenges

**DOI:** 10.1155/2018/4239646

**Published:** 2018-01-30

**Authors:** Semvua B. Kilonzo, Daniel W. Gunda, Bonaventura C. T. Mpondo, Fatma A. Bakshi, Hyasinta Jaka

**Affiliations:** ^1^Department of Internal Medicine, Catholic University of Allied and Health Sciences, P. O. Box 1440, Mwanza, Tanzania; ^2^Department of Internal Medicine, Bugando Medical Centre, P.O. Box 1370, Mwanza, Tanzania; ^3^Department of Medicine, College of Health Sciences, University of Dodoma, P. O. Box 395, Dodoma, Tanzania; ^4^Renal Unit, Department of Internal Medicine, The Aga Khan Hospital, P.O. Box 2289, Dar es Salaam, Tanzania

## Abstract

Hepatitis B is one of the most common infectious diseases in the world with high prevalence in most of sub-Saharan Africa countries. The complexity in its diagnosis and treatment poses a significant management challenge in the resource-limited settings including Tanzania, where most of the tests and drugs are either unavailable or unaffordable. This mini review aims at demonstrating the current status of the disease in the country and discussing the concomitant challenges in diagnosis, treatment, and prevention.

## 1. Introduction

Hepatitis B infection is a disease of global significance affecting large number of people. Chronic Hepatitis B (CHB) infection which embraces a large spectrum of the disease remains to be a serious public health problem globally with over 240 million people being affected and causing 650,000 deaths annually [1].

Hepatitis B virus (HBV) is a small partially double-stranded circular Deoxyribonucleic acid (DNA) virus that belongs to the family Hepadnaviridae. The infectious HBV virion has a spherical, double-shelled structure of about 42 nm in diameter, consisting of a lipid envelope containing Hepatitis B surface antigen (HBsAg). This envelope surrounds an inner nucleocapsid composed of Hepatitis B core antigen (HBcAg) complexed the viral DNA genome. HBV is classified into eight genotypes (A to H), with each one having distinct geographic distribution [2]. The virus is transmitted by exposure to an infected body fluids. Perinatal transmission, and mostly postdelivery, appears to be more common mode than in utero from the mothers with HBsAg positive in high and intermediate HBV endemic zones including Tanzania. Sexual transmission may occur and the most vulnerable group is that of unvaccinated individuals who have multiple sexual partners, men who have sex with men, People Who Inject Drugs (PWID), Healthcare Workers (HCWs), and patients who undergo regular blood transfusions and hemolysis.

Only 5–10% of the infected children develops acute infection while the majority will end up developing the chronic form of the disease [3]. HBV infection embraces wide spectrum of liver diseases including acute and chronic hepatitis, with liver cirrhosis and hepatocellular carcinoma (HCC) being the frequent long-term complications. Following HBV infection, a number of antigens and their corresponding antibodies can be detected in the serum (Table 1). The viral load and liver transaminases can concurrently be distinguished. Therefore the diagnosis of various forms of the disease requires a systematic and integrated interpretation of both virological, serological, clinical, biochemical, and histological findings.

HBV infection is believed to be common in Tanzania where its appropriate diagnosis and conventional management are uncertain. This mini review therefore summarizes the recent information in epidemiology of the disease and the pertaining challenges in its treatment and prevention in the country.

## 2. Epidemiology

The African and Western-Pacific countries harbor 68% of all CHB infections globally [5]. Most of the African countries (99%) are in higher-intermediate and higher-endemic zone with HBsAg seroprevalence of 5–7% and >8%, respectively.

Tanzania is regarded to be a higher-endemic country [6]. A seroprevalence of HBV infection in the country was reported to be 6% in the general population of Dar es Salaam [7]. This rate has increased from older studies that reported the prevalence of 4.4% in the same population [8]. Relatively lower rates of the infection have been seen in children. Muro et al. reported the seroprevalence of 4.2% among children attending different clinics in Kilimanjaro [9], while those of 4.3% and 1.8% were observed in Iringa and Pemba, respectively, among children that were attended in the health facilities for febrile syndromes [10]. The diminished rates of seropositivity in children in particular those who were born from the year 2002 is justifiable as is when the vaccine against HBV was introduced in the country [11], with a persistently satisfactory immunization coverage [12].

### 2.1. Molecular Epidemiology

Accumulating evidence suggests that HBV genotypes may play an important role in the natural course of CHB disease, severity of underlying liver disease, and treatment response [2]. This underlines an importance of identifying the genetic characterization of an infection. Based on nucleotide divergence, 8 genotypes of HBV with several subgenotypes are known to exist, which are distributed in different geographical locations.

In Tanzania, a recent phylogenetic analysis of S-gene sequence revealed HBV genotype A1 (HBV/A1) to be the most common in 86.1% of the study participants. Genotypes D and E were also observed in 12.3% and 0.8% of the individuals, respectively. In this study, blood samples were obtained from individuals residing in several geographical zones of the country (North, East, South, Lake, Zanzibar, and Southern Highland); hence the sample can be generalized to the whole country [13]. The same trend of HBV/A1 predominance was also reported by Hasegawa et al. (90.9%) in Dar es Salaam [14].

An infection with genotype A has frequently been associated with chronicity, better response to interferon therapy, and increased rate of viral resistance during antiviral treatment [2, 15]. In African population, genotype A has interestingly also been found to have a greater potential of hepatocarcinogenicity as compared to other HBV genotypes [15]. These findings are therefore suggesting that majority of Hepatitis B patients in Tanzania will develop chronic infection that is poorly responding to nucleotide analogs antivirals but with a better response to interferons. They are also at increased risk of developing HCC [16].

### 2.2. Blood Donors

The blood donors, which are an eminent population in epidemiology of the disease, appear to have significantly higher rates of HBV markers. According to Tanzania National Blood Transfusion Services (TNBTS) report, HBV infection was the most frequent Transfusion Transmitted Infection (TTI) detected in all blood units collected in the country. The rate of HBV detection was 6.2% and carried 42% of all TTIs. Moreover family replacement donors had excessive risk of being infected by 21.5% as compared to voluntary unpaid donors that was 12.7% [17]. Higher seroprevalence of HBV was also observed among blood replacement donors compared to the voluntary ones in a survey done at Muhimbili National Hospital (MNH) with the rates of 9.5% and 7.2%, respectively [18]. The seroprevalence of 4.8% was reported among the voluntary blood donors in a different study at the same center [19]. Another study that was done in Mwanza, the second largest city of the country, revealed the HBV seroprevalence to be 9.9% among the voluntarily donors and 11.2% in replacement donors [20]. Replacement blood donors, as have been observed in the above studies [17, 18, 20], tend to have higher risk of TTI including Hepatitis B.

### 2.3. Pregnant Women

In the intermediate and high endemic zones of HBV infection where vertical transmission is frequent, pregnant women carry an important role in transmission of a virus. The most recent evidences suggest that intrauterine transmission is rare. It mostly occurs perinatally through direct contact or perfusion of the mother's body fluids to the fetus's circulation by swallowing of an amniotic fluid, contact with vaginal secretion, and the ruptured placenta [21]. Major risk factors for mother-to-child transmission (MTCT) of HBV are high viral load and presence of maternal Hepatitis B envelope antigen (HBeAg) [21–23].

A recent study that involved pregnant women in three antenatal clinics in Dar es Salaam showed the magnitude of HBsAg positivity to be 8% [24]. Much lower prevalence was reported in the previous surveys from Dar es Salaam (3.9%), Kilimanjaro (4.2%), and Mwanza (3.8%). Multigravida women were found to have significantly increased risk of HBsAg positivity as compared to their primigravida counterparts [25–27]. This trend is clearly escalating, suggestive of a need for robust interventions in this particular population. Screening of HBV among pregnant women is not routinely being done in the country.

### 2.4. Healthcare Workers

HCWs are at high risk of acquiring HBV infection due to their frequent occupational contact with infected body materials and contaminated tools. The risk is four times higher than in non-HCWs [27], which increases with prevalence of infection in the general population. On the other hand, the HCWs that have been infected with HBV are similarly at risk of transmitting the infection to their clients, though only rare cases have been reported worldwide [28].

In Tanzania, nearly half of the HCWs have experienced at least one occupational injury in 12-month period [29, 30] that put them in a very high-risk of acquiring Blood-Borne Infections (BBIs). The exact rate of HBV transmission among the HCWs in the country is unknown and many studies have only focused on HIV, which is the only postexposure BBI being frequently screened and prevented in the healthcare settings [31–34]. The only published study in the country that has evaluated the occupational-related HBV status among HCWs reported the seroprevalence of 7%. Majority of these were those considered to have increased risk according to their daily practices. In this study, an advanced age and more duration at work were found to be independently associated with HBV infection. None of the infected HCWs were aware of their infection status [29]. This finding is similar to the one observed in Arusha, where >90% of HCWs in two teaching hospitals were reported not to be aware of their HBV statuses [35].

### 2.5. Patients Undergoing Renal Replacement Therapy

Due to an increased exposure to blood products and shared hemodialysis equipment, patients receiving renal replacement therapy (RRT) are at increased risk of HBV transmission which is one of the major causes of morbidity and mortality among them. These transmissions do not point to inadequacies in the strict infection control guidelines in dialysis but rather to shortcomings in following such recommendations [36].

RRT service is relatively new in Tanzania with only few centers available; therefore, there is paucity of information regarding these patients. There are only two centers (University of Dodoma Dialysis Unit and TMJ Hospital) in the country so far for dialysis patients with HBV infection. The only published data available concerning the HBV status among those patients reported a seroprevalence of 5.2% in Dodoma. In this study, Hepatitis B was found to be a causative agent of kidney disease in 5% of all patients who underwent RRT [37]. Studies showing the prevalence among different population groups in Tanzania have been summarized in Table 2.

## 3. Coinfection with Other Viruses

Due to shared modes of transmission, coinfection of HBV with other viruses, human immune virus (HIV), hepatitis C virus (HCV), and hepatitis D virus (HDV), is likely to occur. When it occurs, it usually triggers an accelerated progression of liver disease with adverse clinical outcomes [47, 48].

### 3.1. HIV

The rates of HBV infection among HIV-infected adults vary considerably in the country. In one meta-analysis review in sub-Saharan Africa (SSA) countries, Tanzania was reported to have coinfection rates ranging 5–17% within different population groups [48]. In the most recent surveys done in Morogoro and Mwanza among ART-naïve HIV-infected patients, HBV/HIV coinfections have been reported to be 6.6% and 7.3%, respectively [42, 43]. This is in keeping with the previous findings from MNH (6.2%) [43]. In both of these studies, younger males with low CD4 count and advanced immune suppression had increased risk of being coinfected. Moreover, the HIV/HBV coinfected patients tend to exhibit rapid clinical deterioration and poor outcomes as compared to their HIV or HBV monoinfected counterparts. These includes high rate of HBeAg seropositivity, increased HBV-DNA, severe hepatotoxicity, occurrence of liver fibrosis, and increased risk of mortality [41, 43, 49]. Relatively lower rates of coinfection have been observed in children (1.2% and 2.9%) [9, 50].

### 3.2. HCV

HCV is known to cause both acute and chronic infections. Usually, 15–45% of acute cases clear the infection without any treatment. Chronic HCV infection is defined as the persistence of the virus in the body for six months or more [50] and is usually determined by the presence of viral antigen and/or DNA in addition to positive serology of Hepatitis C core antibody (HCVcAb). HCVcAb develops during an acute phase and persists throughout the life; therefore, detection of viral antigen/DNA in serological positive individuals is crucial to confirm the presence of virus and hence a need for commencing medication. Serological tests are thereby employed for screening purposes [50].

Few reports that are available in the country on HBV/HCV coinfection by using the screening tests (HCVcAb) are inexplicable. The rates of 5.4% and 3.9% have been seen among PWID and HIV-infected adults, respectively [40, 46], while no cases of coinfection were detected among the blood donors, women in the child-bearing age (15–49 years), and HIV-infected children [9, 18, 25]. The most recent and exclusive study that focused on virological test revealed appalling findings among PWID. Seventy-six percent of serological positive individuals were found to be viremic, implying the presence of chronic active HCV infection, and among them the rate of HBV/HCV coinfection was 9.8% [45]. A fascinating implication from these findings is that nearly one-quarter (24%) of the individuals with positive serology for HCVcAb had actually cleared the infection. These interesting findings are an eye opener, and logically two hypotheses can be derived. First, that the prevalence of Hepatitis C infection is being overestimated in the country due to inability to perform viral confirmatory tests. PWID are regarded worldwide to be the most vulnerable group in HCV transmission [51]. If up to one-quarter of individuals were able to clear an infection (positive HCVcAb, negative DNA), the clearance rate is likely to be elevated in other low-risk population groups despite their HBV statuses. Second, that PWID is the most significant key population in HCV transmission in the country. Supported by high prevalences of HCV monoinfection reported among PWID in Dar es Salaam (27.7%) [52] and Zanzibar (22%) [40], this finding resolves the uncertainty of the previous studies that were unable to divulge the transmission mode of HCV in the country [38, 53].

The standard management of people with HBV/HCV infection that includes the treatment of HCV with antiviral therapy followed by anti-HBV [54] is not being offered in Tanzania, like in other low and middle income countries. This warrants to scale up the preventive measures to reduce the transmission rate. Specific preventive methods recommended by WHO includes screening of the high risk population including PWID, with a subsequent provision of Hepatitis B vaccine, establishment of needle and syringe programs, and psychosocial interventions [55]. Several comprehensive harm reduction programs have been established in the country that are mainly based on psychosocial support with needle and syringe education [52, 56]. Hepatitis B vaccine is still not routinely offered to this particular group.

### 3.3. Other Hepadnaviruses

The occurrence of coinfection of HBV with other forms of hepatitis has rarely been reported in the country. Available literatures have shown the infection rates of HAV to be 0.8% among HIV-infected adults attending a clinic in MNH [46], while none of the HDV was detected among HIV cohort in rural Tanzania [57]. Low prevalence of HEV monoinfection was seen in one study (0.2%) with no coinfection with HBV [7]. The presence of these infections, however, in the neighboring countries imply the possibility of having substantial number of undiagnosed cases in Tanzania, considering a similar presentation of acute hepatitis in both HAV, HDV, and HEV as that of HBV infection [58, 59]. Further studies are therefore warranted in this area.

## 4. Treatment

**Figure 1:**
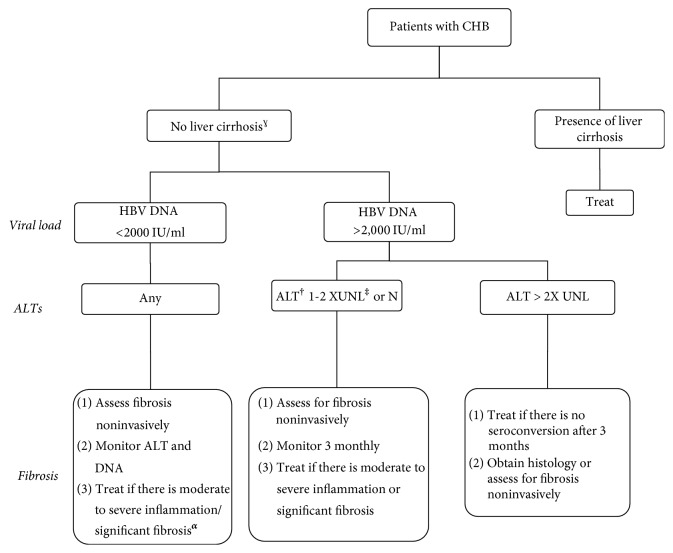
Indications for starting the treatment in patients with chronic hepatitis B virus infection [1]. ^†^ALT: Alanine Aminotransferase. ^‡^UNL: upper normal limit. ULN is 30 U/L for men and 19 U/L for females. ^*α*^Significant  fibrosis is defined by liver stiffness ≥ 8 kPa (by Fibroscan) or [AST]-to-platelet ratio index (APRI) ≥ 1.5. ^**ɣ**^Liver cirrhosis is determined by liver stiffness ≥ 11 kPa (by Fibroscan) or APRI ≥ 2.0.

Indications for HBV treatment are strict, as it has to be offered only to CHB patients with inflammation of the liver, fibrosis, high viral replication, and/or at high risk of disease progression to cirrhosis or HCC. A patient is therefore entailed to undergo a bunch of serial investigations prior to the commencement of treatment. The details of these indications have been shown in Figure 1. To date, seven antiviral agents (Lamivudine, Adefovir, Entecavir, Telbivudine, Tenofovir, Emtricitabine, and Standard and Pegylated Interferon) have been approved for the treatment of CHB [1]. Suppression of HBV-DNA to undetectable level with a serological response for HBsAg and HBeAg (in HBeAg-positive) is a desired goal for CHB treatment, and most guidelines recommend this to be achieved within 48 weeks of treatment.

Unavailability of most these tests and drugs in the country poses difficulty in decision-to-treat and subsequent follow-up of management. The only widely available potent anti-HBV drug in the country is Tenofovir, which is usually supplied in a combination therapy with other antiretro viral drugs that is deemed for treatment of HIV infection. This creates a challenging situation in management of HBV monoinfected cases. HBV in monoinfected and in HIV coinfected patients is known to have extensive resistance to Lamivudine [60, 61], which is still being recommended and used in some areas of Tanzania [62]. To date, there is only one treatment center in the country where Tenofovir monotherapy is being provided to HBV monoinfected patients.

## 5. Prevention

Available anti-HBV drugs cannot eradicate the virus; hence a lifelong treatment is usually required. The regular availability of these drugs in most of low and middle income countries is also a problem. This necessitates the scaling up of preventive measures especially in the population that is at high risk of infection.

### 5.1. HBV Vaccination

An effective vaccine against HBV has been available since 1982, and it has been included in the pediatric vaccination programme since 2002 in the country. The vaccine is being offered during the 4th, 8th, and 12th weeks after delivery. As most of HBV transmission in intermediate and high endemic regions occurs within first 5 years of life, effective neonatal vaccination is essential as it confers immunity in 80–95% of the cases [63]. Lower protective rate has been reported to occur in Tanzanian children where only 70% of the vaccinated ones (less than 5 years old) were able to mount a protective level of immunity (anti-HBs > 10 I/U). HIV status and the number of doses were the significant predictors of immunity levels in this study [64]. Similar findings were reported in another study in Dodoma with the protective rate of only 59.5% among HIV-infected children. In this survey, elevated CD4 count and the use of ARTs were independent predictors of successful immunization [65]. The diminished protective rate of vaccine in Tanzania might be influenced by a delayed impact of the first dose of the vaccine that is usually given at the 4th week, where an infection is probably likely to have already occurred. The recommended practice is offering of the first dose of vaccine immediately after birth or within 24 hours after delivery [1, 66–68]. As a preventive measure, HBV vaccine is also recommended in other high-risk population groups like HCWs, PWID, HCV infected patients, and sex workers (in low infant immunization coverage areas). A routine vaccination to these groups is not been offered in the country.

### 5.2. Prevention of Mother-to-Child Transmission

In its Global Health Sector Strategy on Viral Hepatitis (2016–2021) for elimination of Hepatitis B by 2030, WHO recommends a comprehensive approach in reduction of MTCT. These include routine HBV screening with a subsequent treatment of pregnant women diagnosed to have HBV infection, providing HBV vaccine to an infant within 24 hours of birth, safe delivery practices, and the development of new interventions based on maternal antiviral treatment [69]. Short-term maternal antivirals (Tenofovir or Telbivudine) given during the second or third trimester of pregnancy in addition to HBV vaccine and Hepatitis B Immunoglobulin (HBIG) to the newborn baby have been shown to decrease perinatal MTCT in several trials [68, 70]. Various countries have already adapted this regime [63, 67, 71–73].

Despite this call, these strategies have not yet been introduced in Tanzania. Most of antenatal care centers in the country have not yet integrated their services to accommodate the care for HBV-infected patients. The transmission rates of HBV from mother to child also is not known.

### 5.3. Prevention in Healthcare Setting

#### 5.3.1. Blood Transfusion

Median overall risk of becoming infected with HBV from blood transfusions in SSA is estimated to be 4.3 per 1000 units of blood. This is largely contributed by incomplete screening of TTI in transfused blood [74]. The blood safety standards include collection of blood from voluntary nonremunerated blood donors from low-risk populations of TTI in a nationally coordinated blood transfusion service and screening of donated blood for all TTIs [75].

Before the establishment of NBTS in the country in 2004, family donation was the significant source of the transfused blood that was mostly used for replacement purposes [76]. To date, about one-third of the blood that is being transfused is being issued by NBTS blood banks, which is usually rigorously screened and tested for all TTIs including Hepatitis B [77]. The plans are underway to scale up the NBTS services to cover the larger part of the country.

#### 5.3.2. Healthcare Workers

Due to their occupational increased risk of contracting HBV, screening and vaccination to all HCWs to maintain an anti-HBs > 10 mIU/mL are recommended and for those who are HBsAg positive a potent antiviral agent should be provided to maintain the HBV-DNA < 2000 IU/mL. In addition, a postexposure prophylaxis of HBIG with active vaccination (in nonimmune) has to be commenced immediately following an occupational hazard [63, 66, 67, 72, 73].

The vaccination status among the HCWs against HBV is poor with only 48.8% of the HCWs in a referral hospital in Mwanza reporting to have received 3 doses of vaccine within ten years. Of these, 23% were unable to mount the immunity (anti-HBs titre < 10 ml/U), leaving them in a risk of acquiring HBV infection [29]. The same trend has also been observed in Arusha where 89% of HCWs reported not to have received any HBV vaccine, the main reason being unawareness of the vaccine [35].

Despite having substantial number of HCWs who are at risk of acquiring occupational HBV infection, most of these preventive measures are not in place in the country. A formal vaccine for HBV is only being offered to the infants as a part of the country's vaccination program, and HBIG is not readily available [11].

#### 5.3.3. Injection Safety, Sharps, and Waste Management

The Ministry of Health, Community Development, Gender, Elderly and Children (MoHCDGEC) has published several guidelines on IPC in which extensive instructions on proper hand washing, surgical hand preparations, use of gloves, injection safety, safe cleaning of equipment, and sharps disposal have been provided [78]. In spite of this, the knowledge and practice of these safety measures among HCWs vary considerably in the country with excellent performances being observed in some areas [79, 80], while others exhibit substandard practice [81, 82]. All in all, in order to achieve consistently safe practices, continuous training on IPC in HCWs is necessitated.

## 6. The Government's Efforts to Control HBV Infection

Despite several incongruities in the Tanzanian health system in almost all aspects regarding the imminent HBV infection, the government is taking appropriate measures to address these constrains. It has already been announced that HBV vaccines will be freely offered to the high-risk groups from the year 2018 [83].

Moreover, a comprehensive 5-year HBV treatment plan has already been launched, using MNH as a pilot with the future plans of extension to cover the whole country [84].

In addition, the government is scaling up the blood safety program by expanding the NBTS centers. On top of the available zonal NBTS centers, nine regional NBTS stations have already been constructed to date and an expansion to the remaining regions is on the way.

Also, in line with the recommendations from WHO towards elimination of Hepatitis by 2030, the high level national committee for hepatitis elimination program has already been formed in the country and currently the government is setting up the national elimination targets and the strategic plans. The subsequent step will be the dedication of funds for the universal access for treatment. In recognition of these efforts, the country has been recently attributed by WHO to be among the countries that are significantly advancing in the efforts to eliminate viral hepatitis [85].

## 7. Conclusions and Recommendations

HBV infection is common in Tanzania. A number of challenges exist in almost all aspects pertaining to the care of the disease. Besides the government's efforts to resolve these glitches, virtuous achievements will only be accomplished when several epidemiological and clinical research gaps of the disease have been filled. These include true prevalences of different clinical forms of the disease, mother-to-child transmission rates, feasibility of using readily available noninvasive tools for diagnosis of liver fibrosis/cirrhosis, cost-effectiveness of several anti-HBVs with their local resistance patterns, and an existence of other hepadnaviruses (A, D, C, and E) that usually accelerate the clinical downfall in coinfected patients with HBV.

## Figures and Tables

**Table 1:** Interpretation of Hepatitis B results [4].

Component	Test results	Interpretation
HBsAg	Negative	Susceptible to infection
Anti-HBc	Negative	
Anti-HBs	Negative	

HBsAg	Negative	Immune due to previous infection
Anti-HBc	Positive	
Anti-HBs	Positive	

HBsAg	Negative	Immune due to Hepatitis B vaccination
Anti-HBc	Negative	
Anti-HBs	Positive	

HBsAg	Positive	Acute hepatitis B infection
Anti-HBc	Positive	
IgM anti-HBc	Positive	
Anti-HBs	Negative	

HBsAg	Positive	Chronic Hepatitis B infection
IgG anti-HBc	Positive	
IgM anti-HB	Negative	
Anti-HBs	Negative	

HBsAg	Negative	Either: (1) Resolved infection (2) “Low level” chronic infection (3) Resolving acute infection
Anti-HBc	Positive	
Anti-HBs	Negative	

HBeAg	Positive	Presence of active HBV replication and high infectivity

HBsAg	Negative	Occult HBV infection
Anti-HBc	Positive/Negative	
HBV DNA	Positive	

**Table 2:** Hepatitis B prevalence in Tanzania among different population groups.

Population type		Study	Year	Region/city	Prevalence
All age groups		Pellizzer et al. [8]	1994	Dar es Salaam	4.4%
Adults		Miller et al. [7]	1998	Dar es Salaam	6.0%
Children		Muro et al. [9]	2013	Kilimanjaro	4.2%
Children		Meschi et al. [10]	2010	Iringa	4.3%
Children		Meschi et al. [10]	2010	Pemba	1.8%
Blood donors	All	MoHCDGEC [17]	2016	Nationwide	6.2%
	All	Hasegawa et al. [14]	2006	Dar es Salaam	4.8%
	All	Matee et al. [38]	1999	Dar es Salaam	11%
	All	Jacobs et al. [20]	1997	Mwanza	11.2%
	Replacement donors	Matee et al. [18]	2006	Dar es Salaam	9.5%
	Voluntary donors	Matee et al. [18]	2006	Dar es Salaam	7.2%
Health care workers		Mueller et al. [29]	2015	Mwanza	7.0%
Pregnant women		Manyahi et al. [24]	2017	Dar es Salaam	8.0%
		Mirambo et al. [26]	2016	Mwanza	3.8%
		Rashid et al. [39]	2014	Dar es Salaam	3.9%
		Msuya et al. [25]	2006	Kilimanjaro	4.2%
		Pellizzer et al. [8]	1994	Dar es Salaam	4.3%
PWID		Mohammed et al. [40]	2006	Zanzibar	2.1%
Coinfections					
HBV/HIV	Adults	Kilonzo et al. [41]	2017	Mwanza	6.6%
	Adults	Ramírez-Mena et al. [42]	2016	Morogoro	7.3%
	Adults	Hawkins et al. [43]	2013	Dar es Salaam	6.2%
	Children	Muro et al. [9]	2013	Kilimanjaro	2.9%
	Children	Telatela et al. [44]	2007	Dar es Salaam	1.2%
HBV/HCV	PWID	Mohammed and Salim [45]	2017	Dar es Salaam	9.8%
	HIV-infected adults	Nagu et al. [46]	2008	Dar es Salaam	3.9%
	PWID	Mohammed and Salim [40]	2006	Zanzibar	5.4%
HEV	Adults	Miller et al. [7]	1998	Dar es Salaam	0%

## List of Abbreviations

BBIs: Blood-borne Infections
CHB: Chronic Hepatitis B
DNA: Deoxyribonucleic acid
HBeAg: Hepatitis B envelope antigen
HBIG: Hepatitis B Immunoglobulin
HBcAg: Hepatitis B core antigen
HBsAg: Hepatitis B surface Antigen
HBV: Hepatitis B virus
HCC: Hepatocellular carcinoma
HCVcAb: Hepatitis C virus core antibody
HCV: Hepatitis C virus
HCWs: Healthcare Workers
HDV: Hepatitis D virus
HEV: Hepatitis E virus
HIV: Human immune virus
IPC: Infection Prevention and Control
MNH: Muhimbili National Hospital
MoHCDGEC: Ministry of Health, Community Development, Gender, Elderly and Children
MTCT: Mother-to-child transmission
PWID: People Who Inject Drugs
RRT: Renal Replacement Therapy
TNBTS: Tanzania National Blood Transfusion Services
TTI: Transfusion Transmitted Infection
WHO: World Health Organization.
